# Supplementary material for: Astrocyte–neuron subproteomes and obsessive–compulsive disorder mechanisms
Source: Nature. 2023 Apr 12;616(7958):764–73. doi: 10.1038/s41586-023-05927-7 (PMC10132990; doi:10.1038/s41586-023-05927-7)

nature portfolio

https://doi.org/10.1038/s41586-023-05927-7

Supplementary information

Astrocyte–neuron subproteomes and obsessive–compulsive disorder mechanisms

In the format provided by the authors and unedited

Astrocyte-neuron subproteomes and obsessive-compulsive disorder mechanisms

Joselyn S. Soto, Yasaman Jami-Alahmadi, Jakelyn Chacon, Stefanie L. Moye, Blanca Diaz-Castro, James A. Wohlschlegel & Baljit S. Khakh

Supplementary Figure 1

Full western blots

Corresponding to Extended data fig 2e

Streptavidin HRP

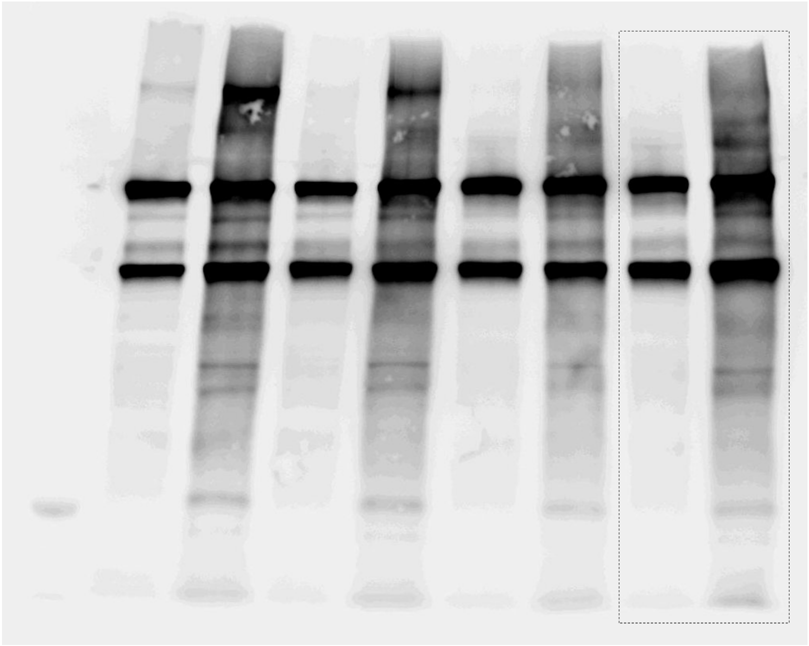

Beta-actin

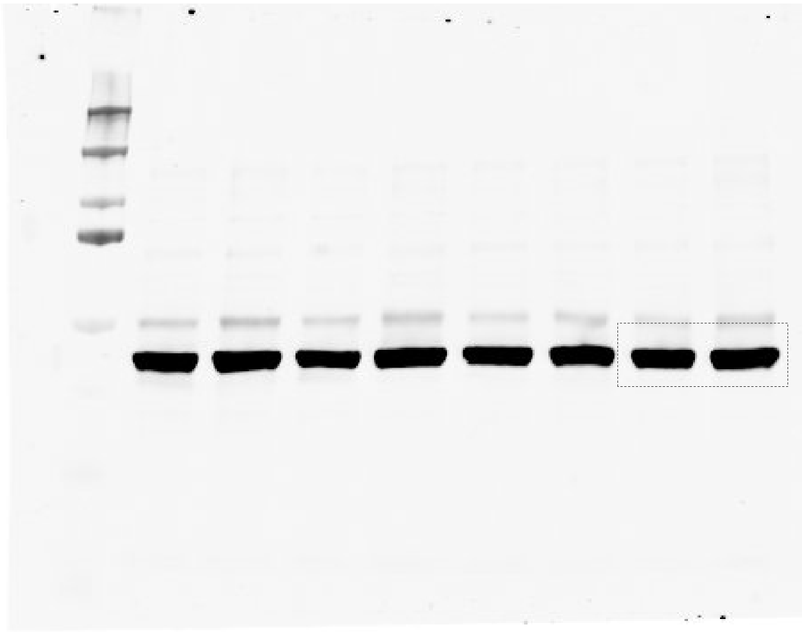

Note: the brightness/contrast were increased in all the blots here to show the edge of the blot as best as possible without overexposing.
Both images represent the same blot. Left images show chemiluminescence signal, while right blots show fluorescence as stated in the methods.

Corresponding to Extended data fig 2f

Streptavidin HRP

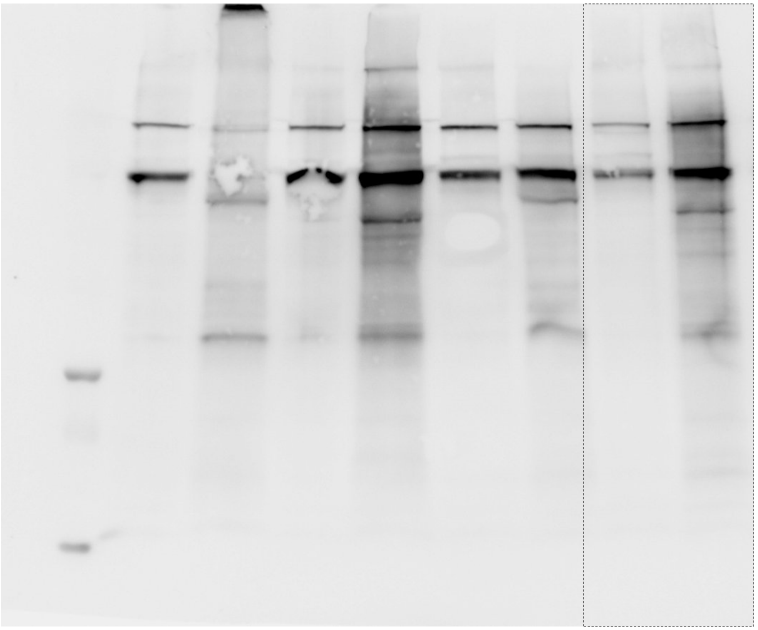

Beta-actin

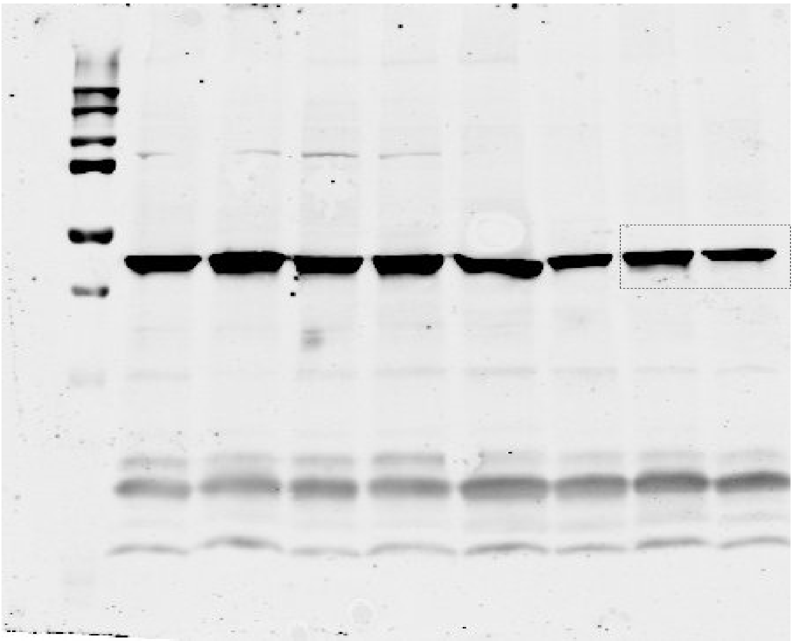

Corresponding to Extended data fig 2g

Streptavidin HRP

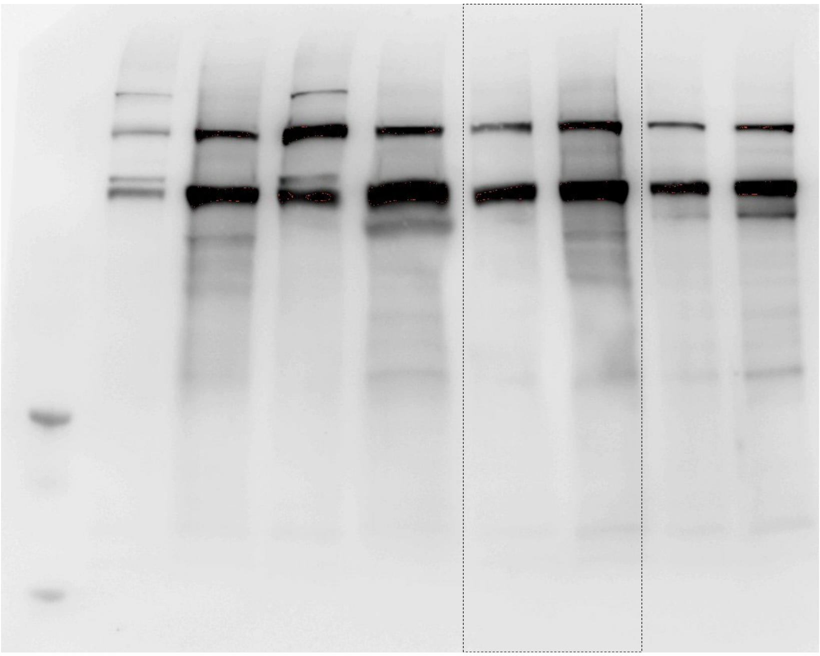

Beta-actin

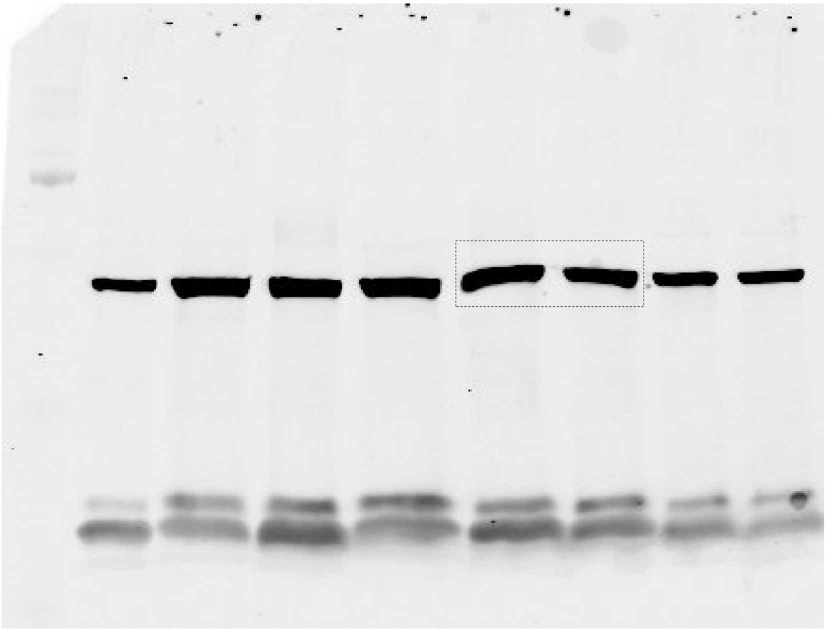

Corresponding to Extended data fig 2h

Streptavidin HRP

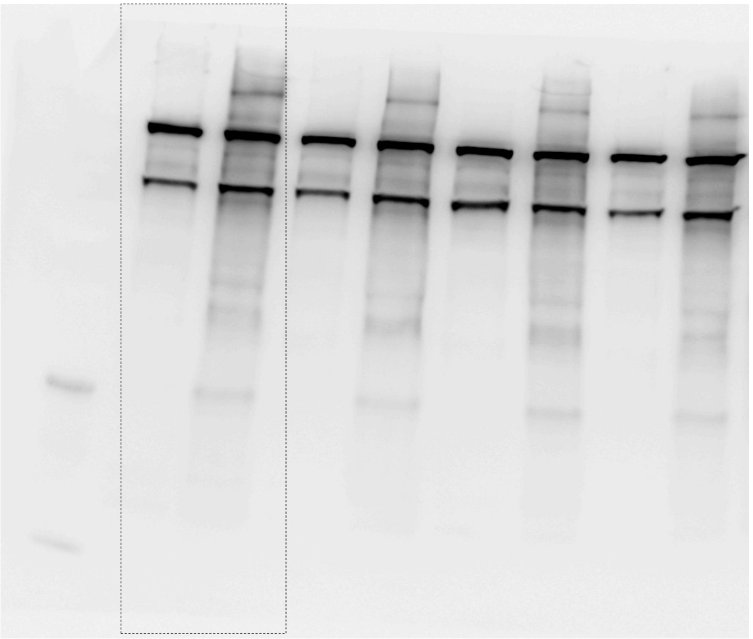

Beta-actin

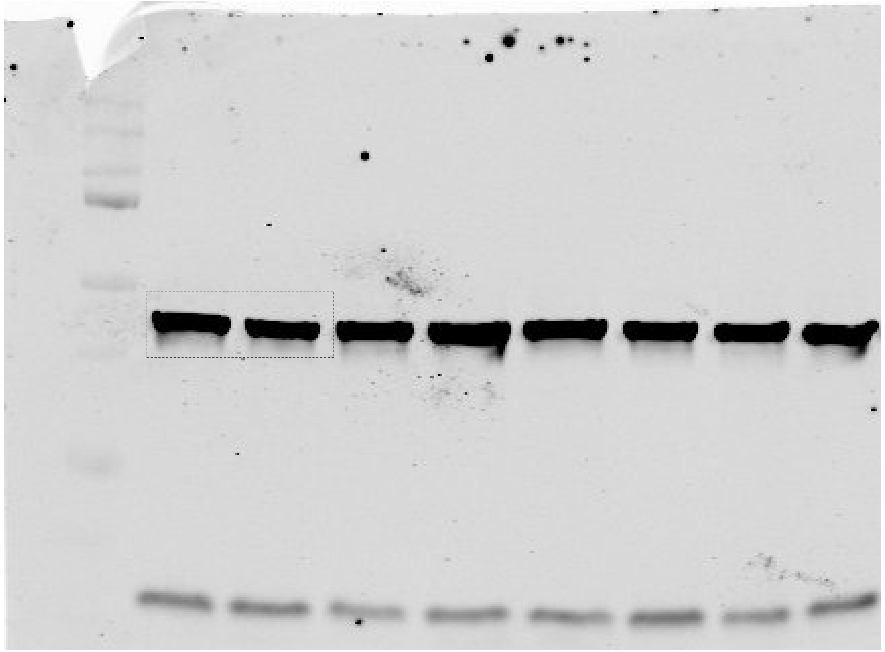

Corresponding to Extended data fig 3c

Streptavidin HRP

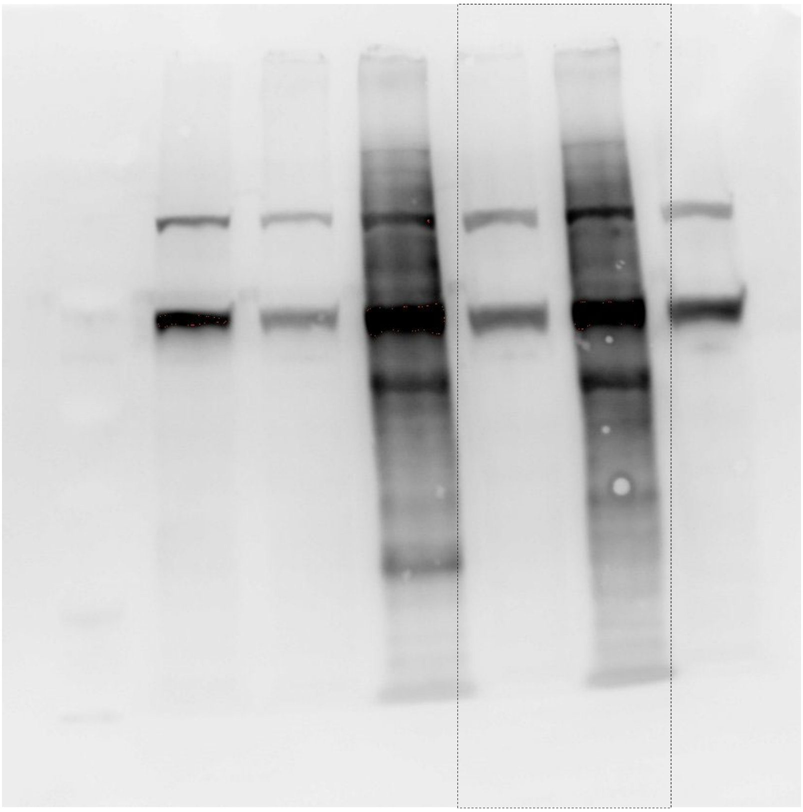

Beta-actin

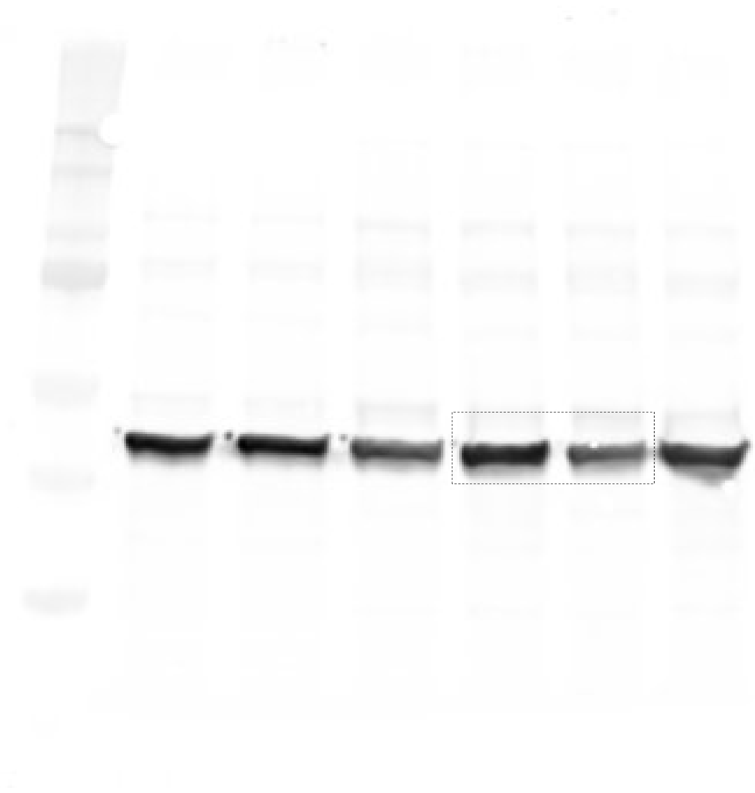

Corresponding to Extended data fig 3g

Streptavidin HRP

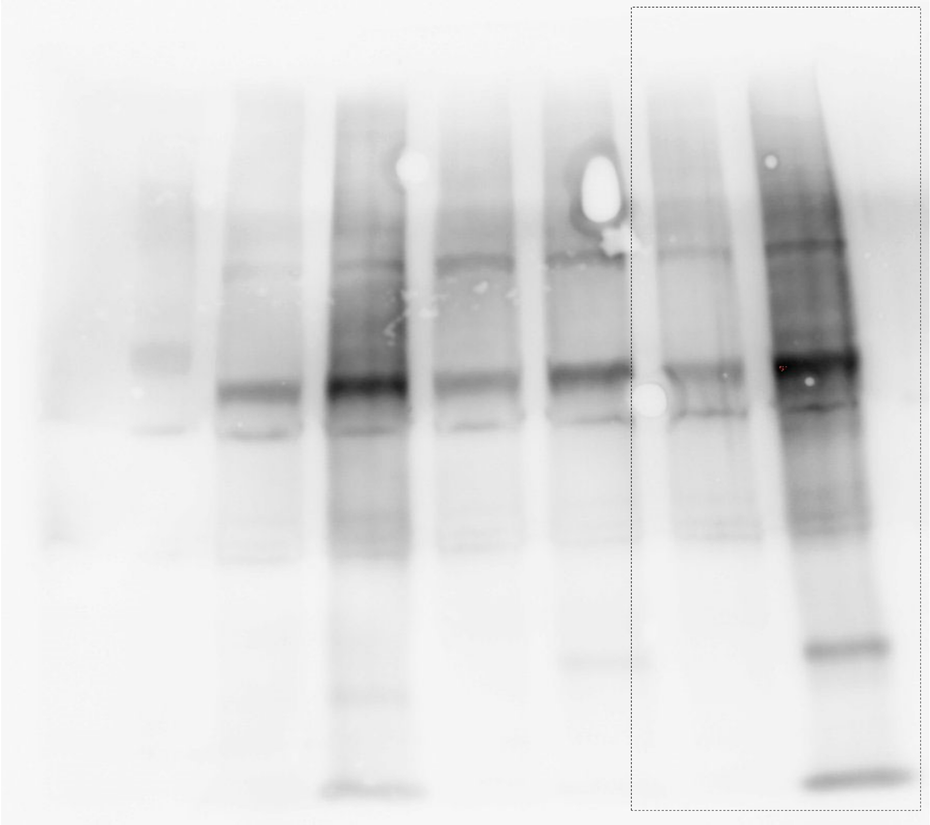

Beta-actin

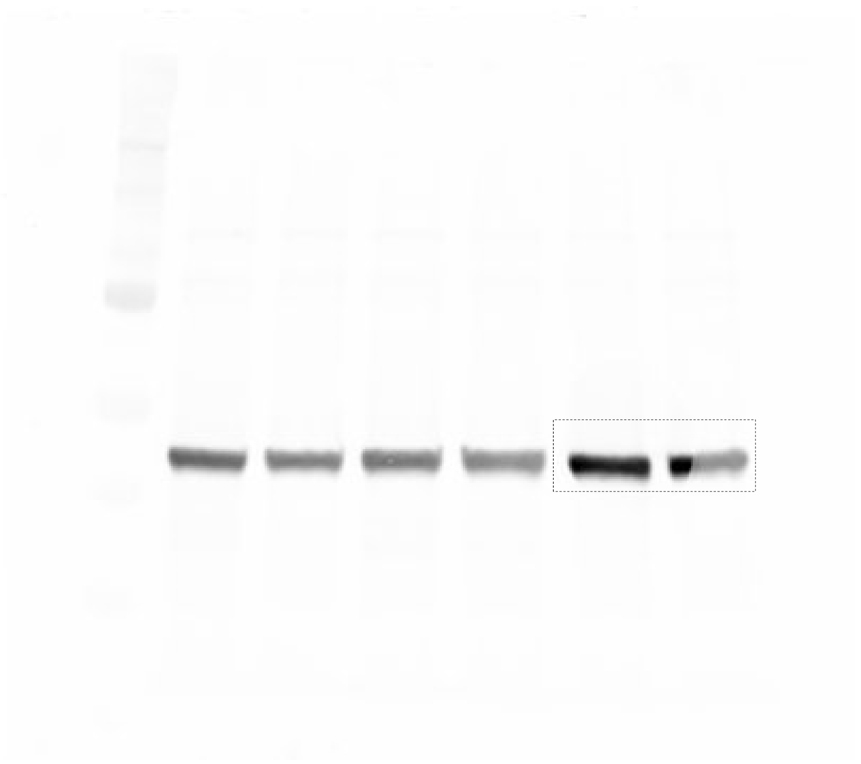

Corresponding to Extended data fig 10a

Streptavidin HRP

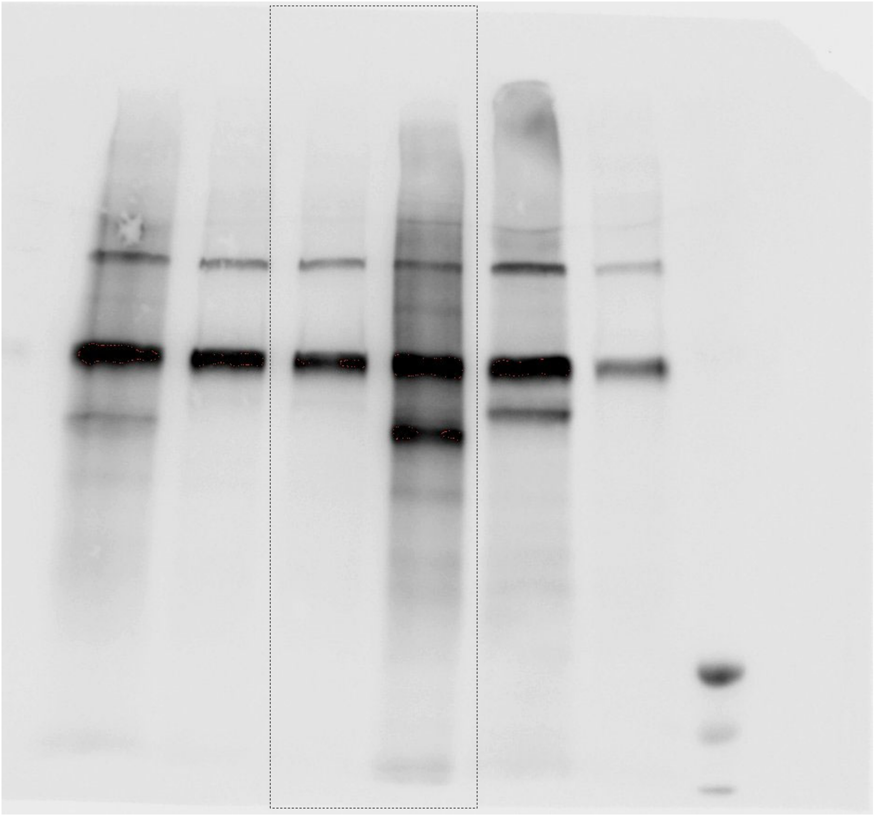

Beta-actin

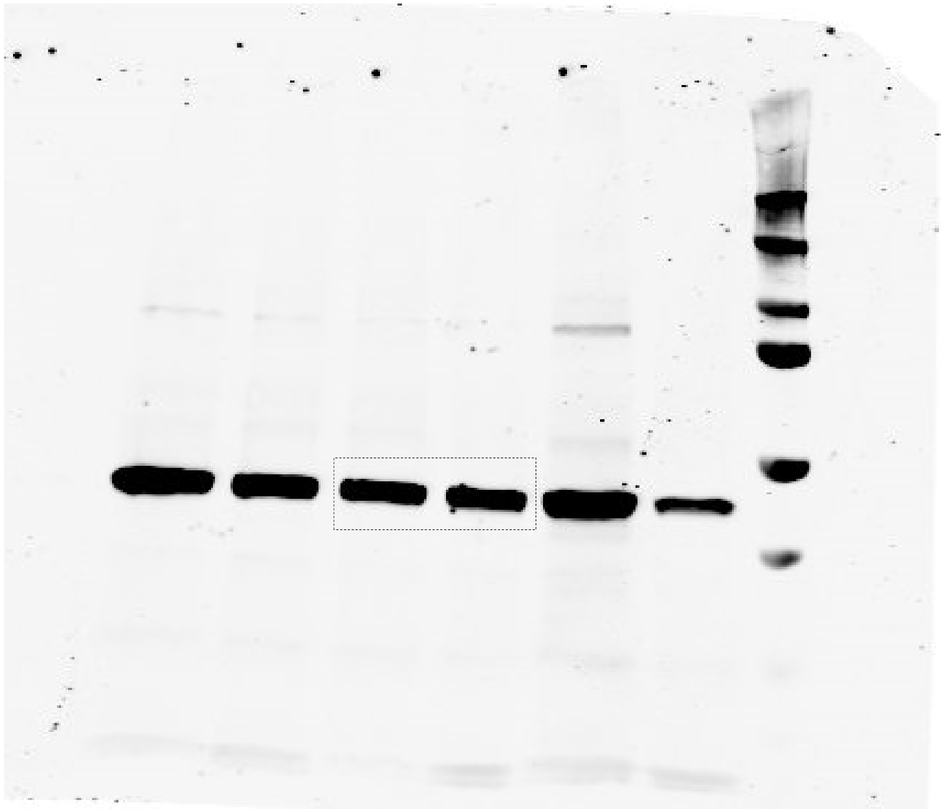

Corresponding to Extended data fig 10b

Streptavidin HRP

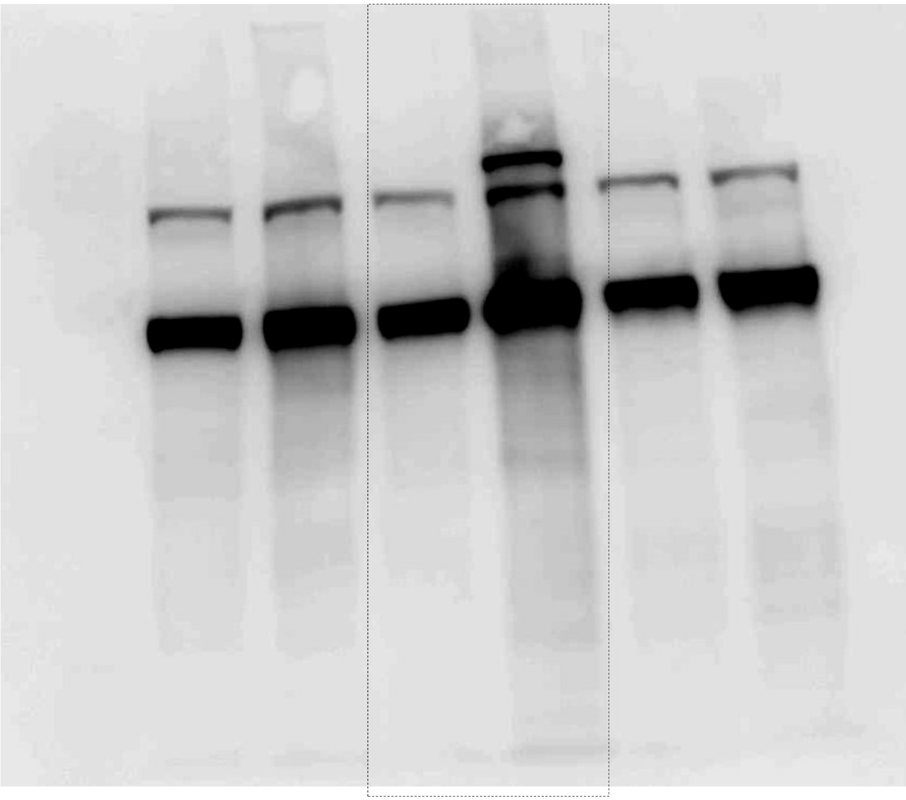

Beta-actin

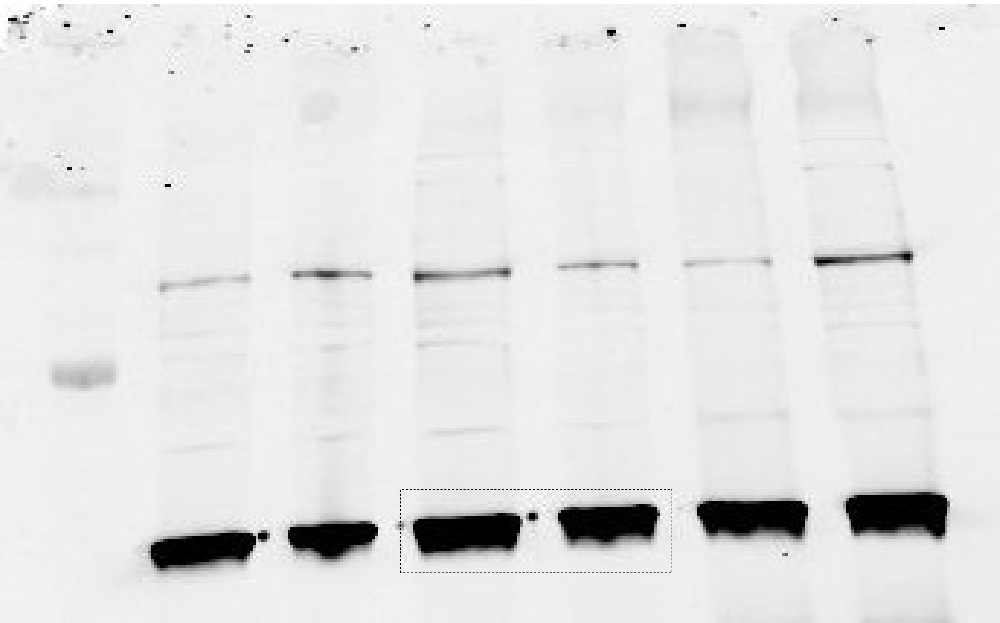

Corresponding to Extended data fig 10c

Streptavidin HRP

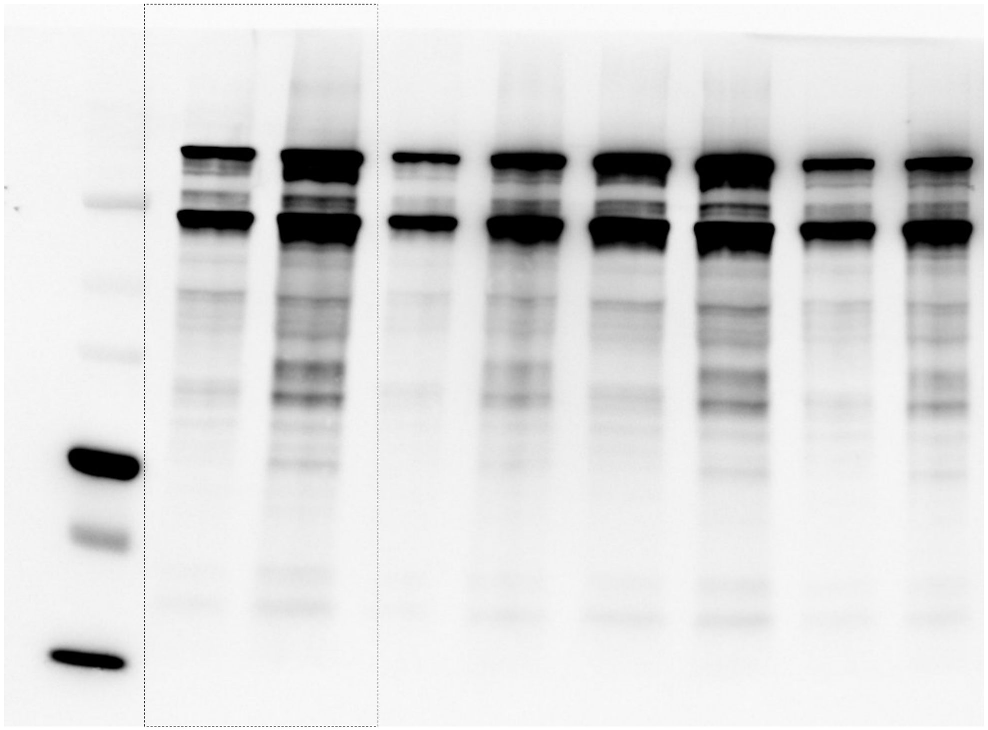

Beta-actin

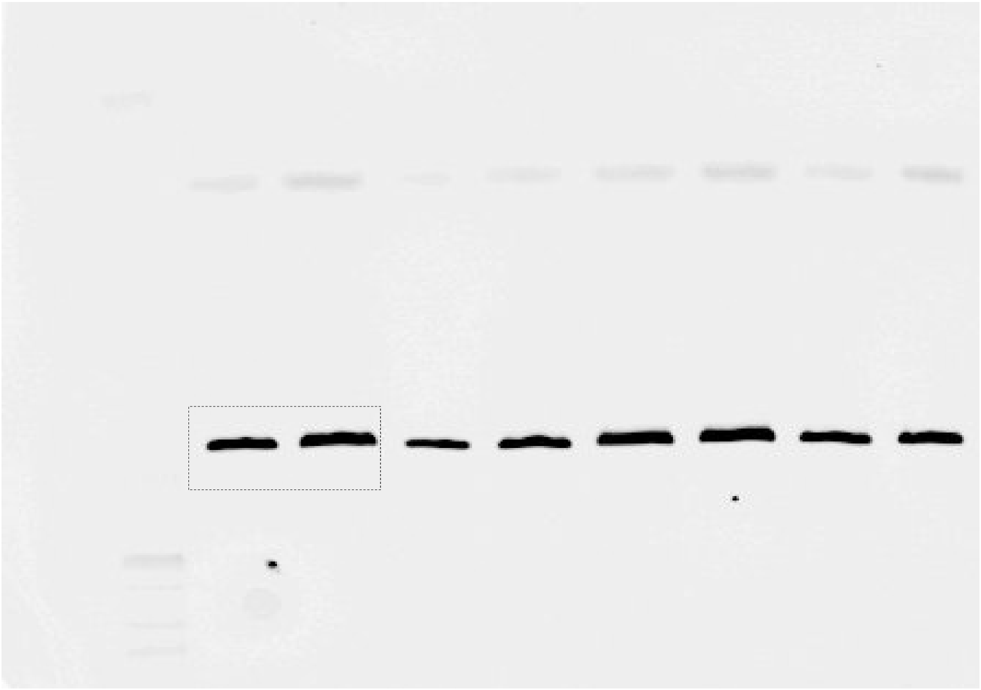

Corresponding to Extended data fig 10d

Streptavidin HRP

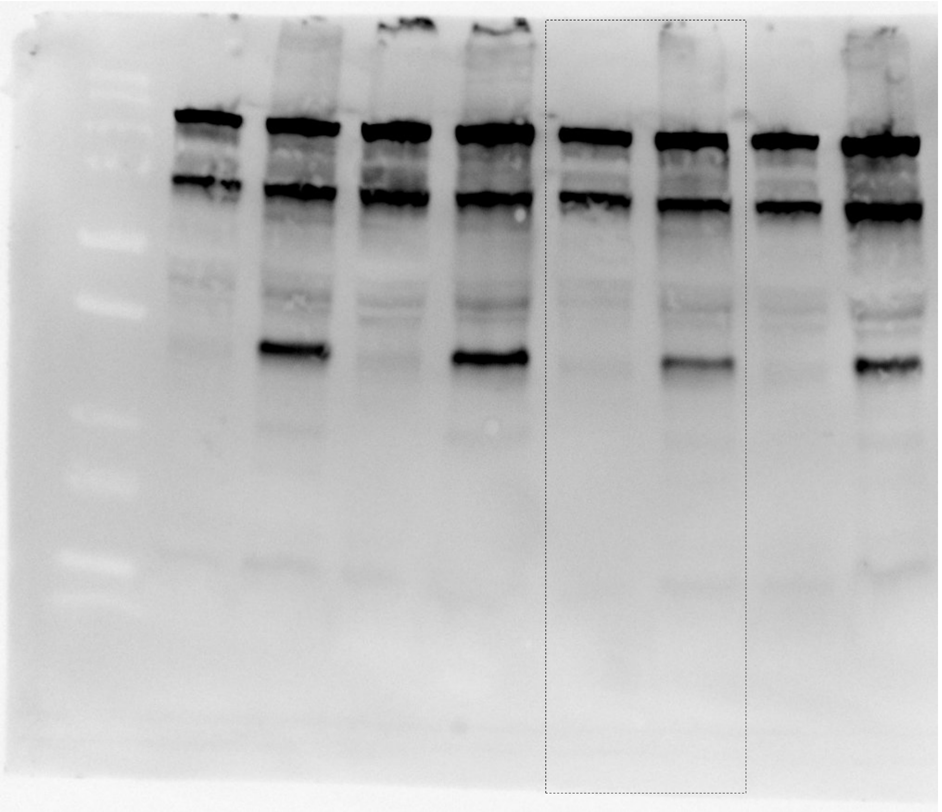

Beta-actin

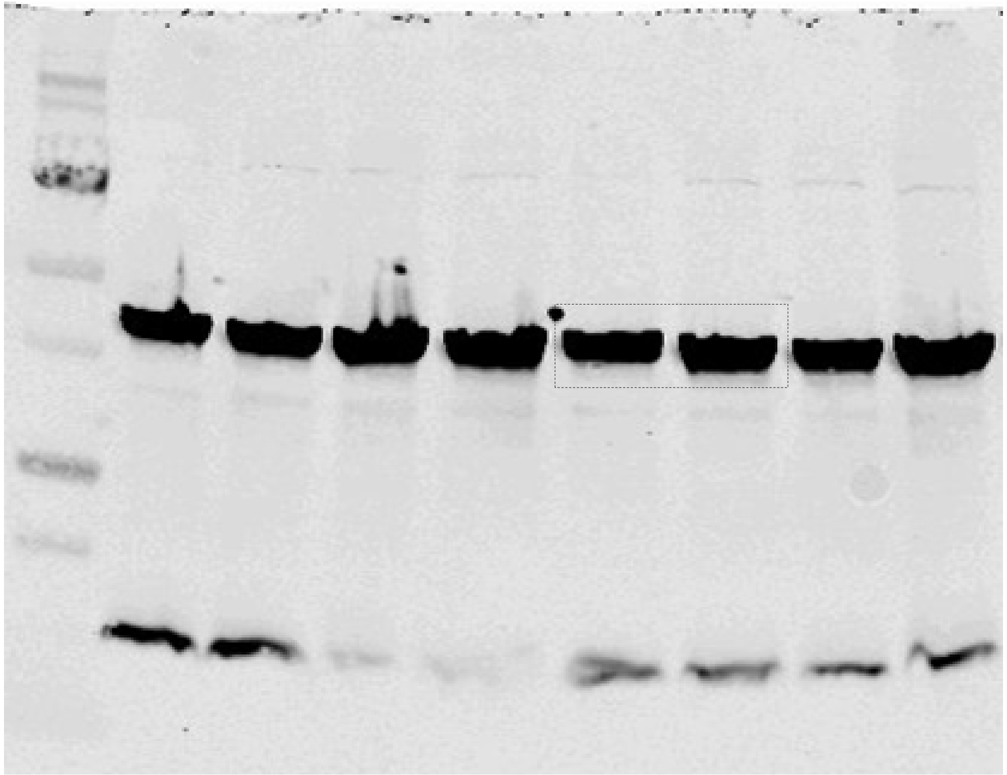

Corresponding to Extended data fig 10e

Streptavidin HRP

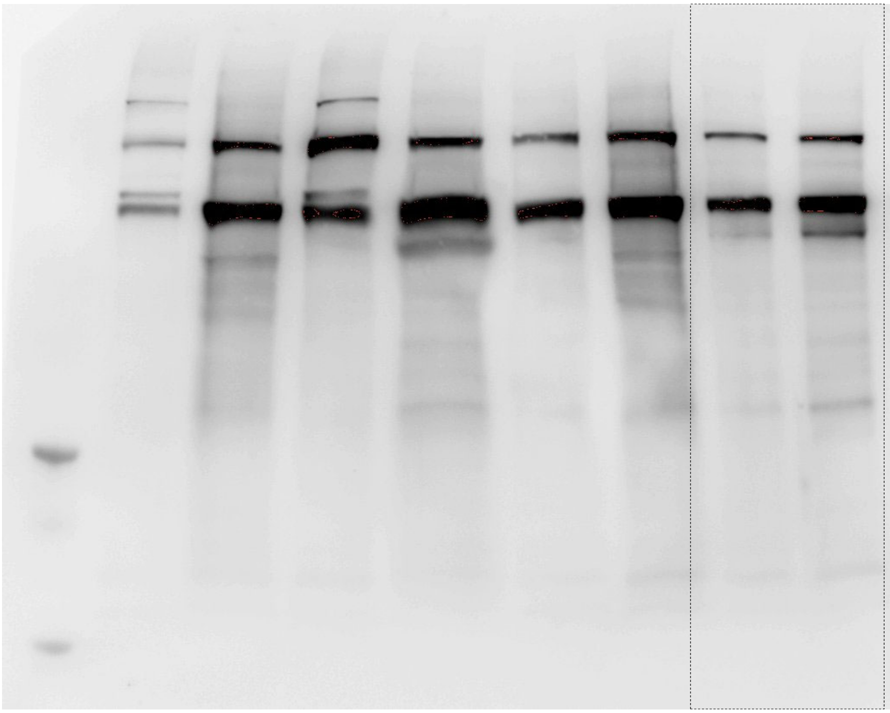

Beta-actin

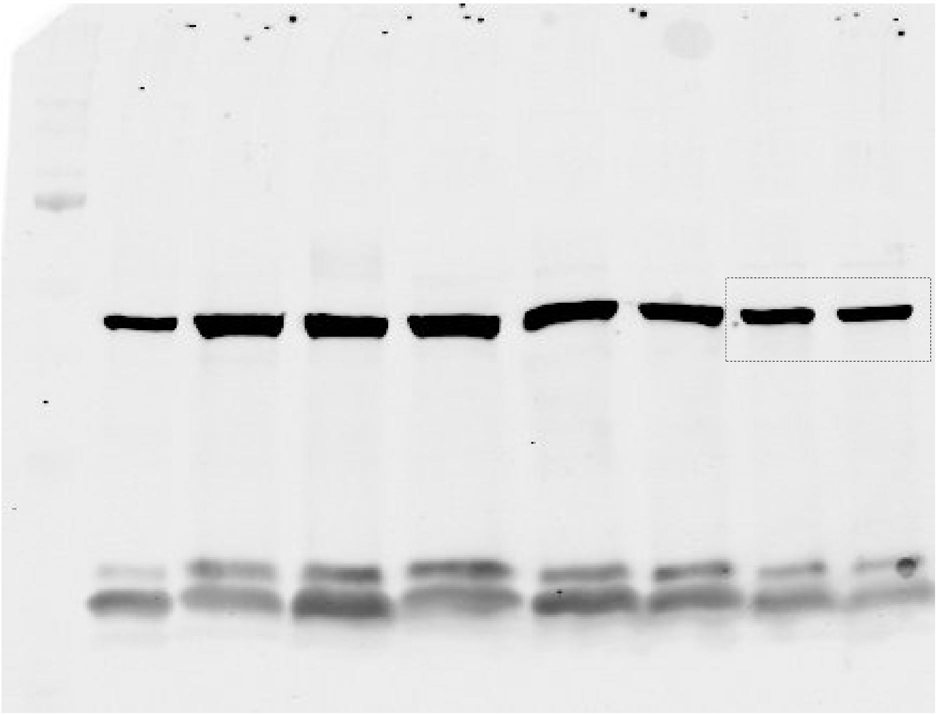

Corresponding to Extended data fig 17e

Streptavidin HRP

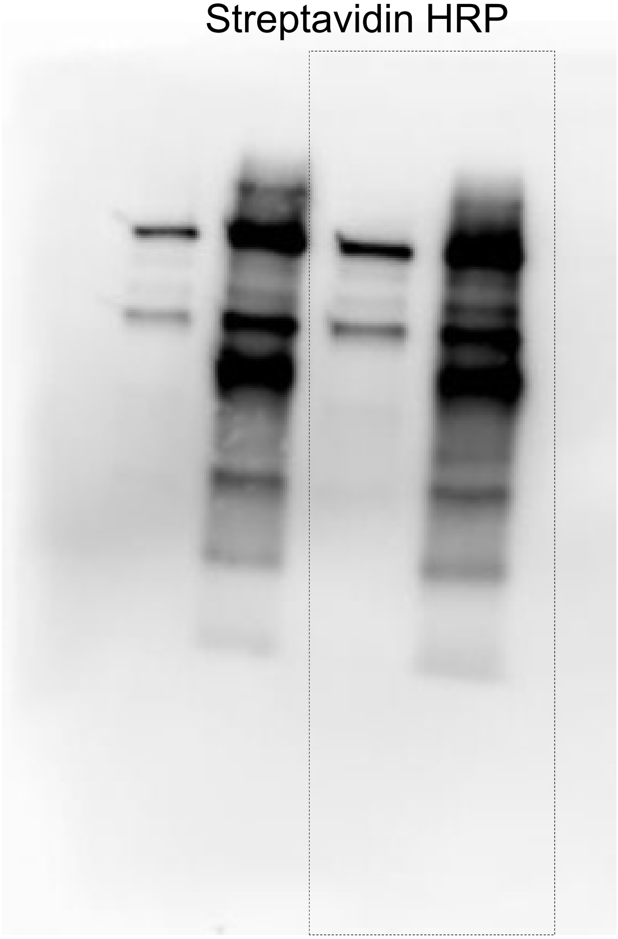

Beta-actin

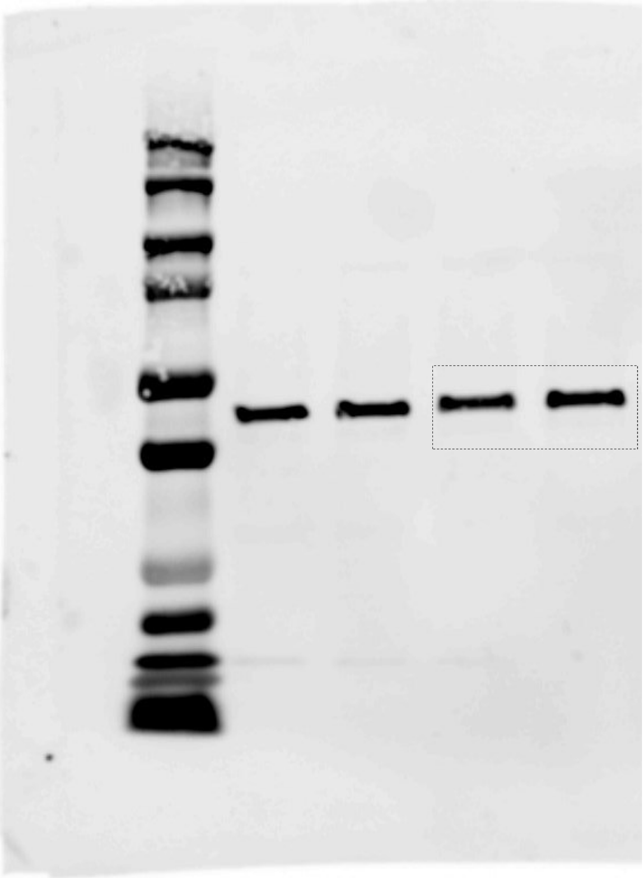

Corresponding to Extended data fig 17f

Streptavidin HRP

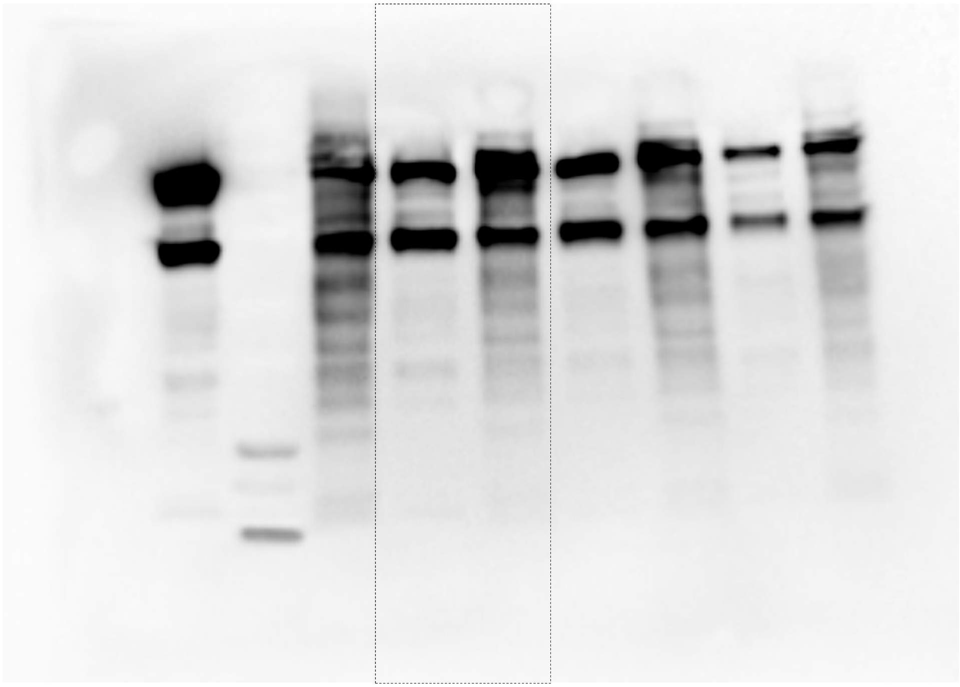

Beta-actin

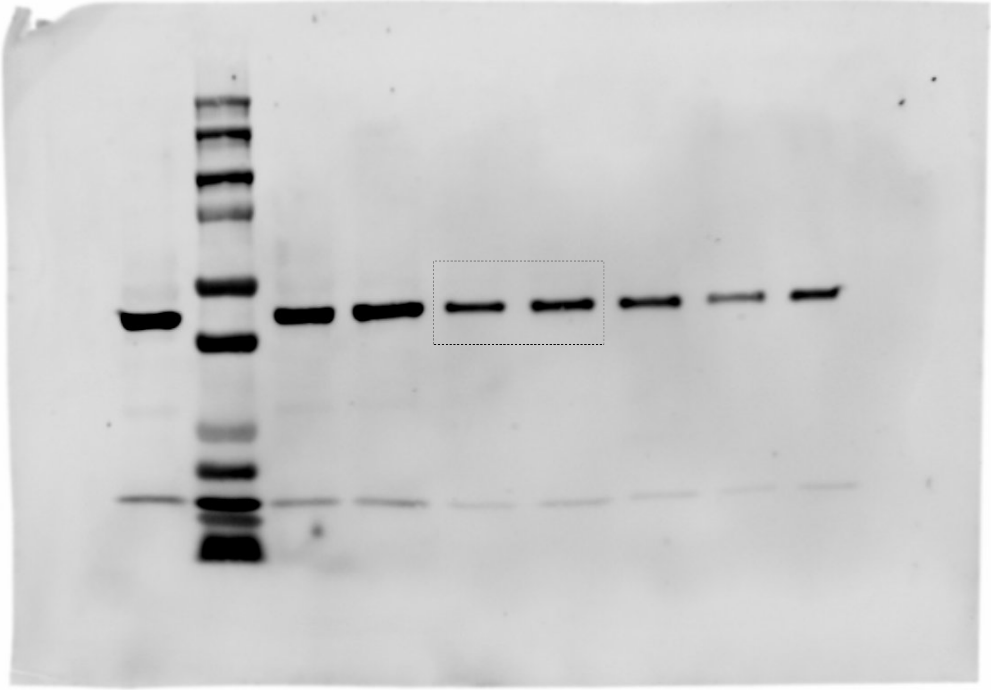

Corresponding to Extended data fig 18d

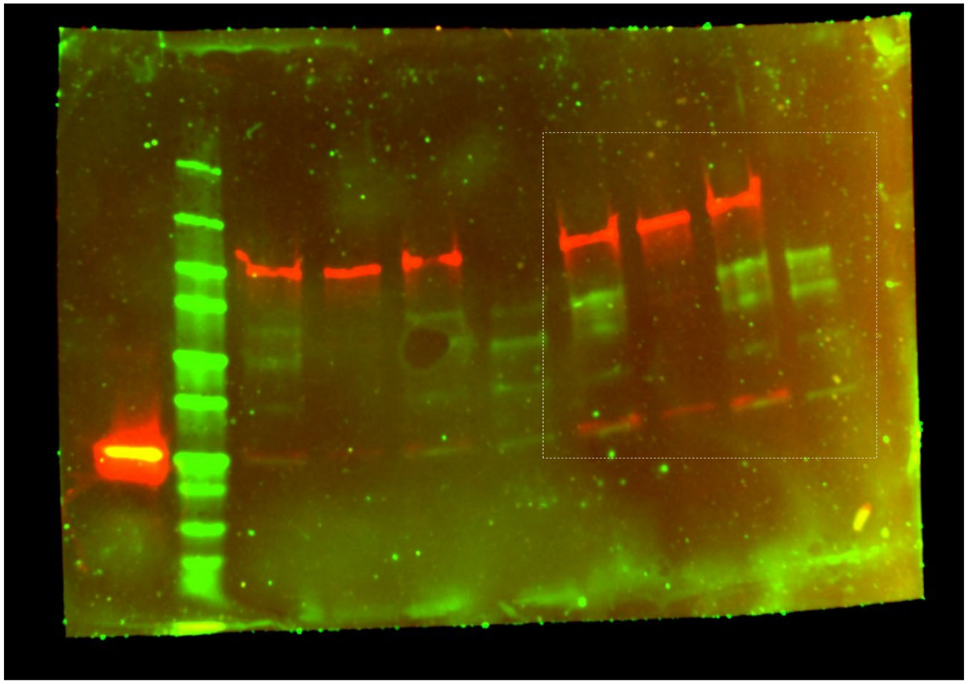

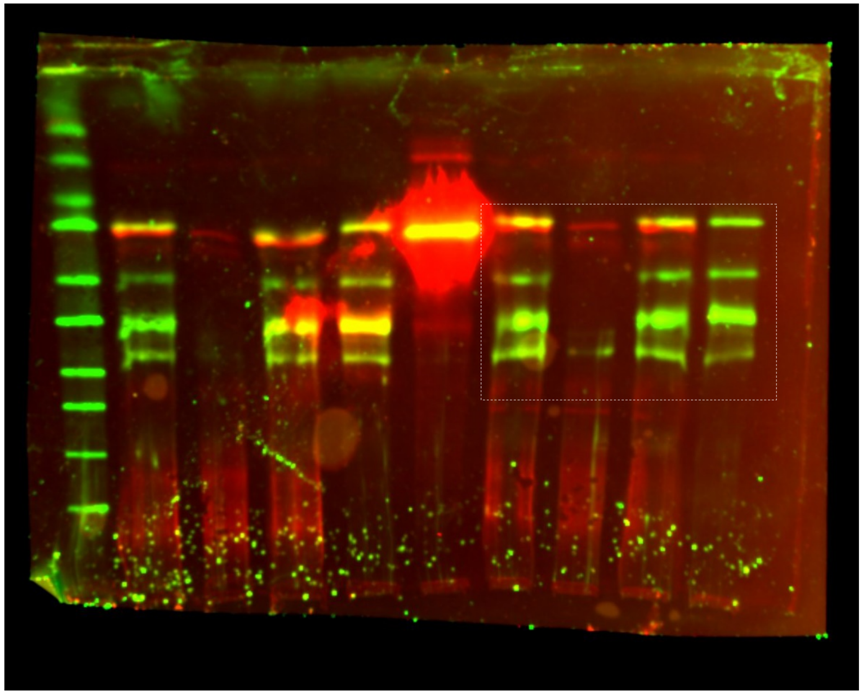

Supplement: Supplementary file 1 — Raw source blots for western blots. [file 41586_2023_5927_MOESM1_ESM.pdf]
