# Supplementary material for: Astrocyte–neuron subproteomes and obsessive–compulsive disorder mechanisms
Source: Nature. 2023 Apr 12;616(7958):764–73. doi: 10.1038/s41586-023-05927-7 (PMC10132990; doi:10.1038/s41586-023-05927-7)
Supplement: Supplementary file 2 — Reporting Summary [file 41586_2023_5927_MOESM2_ESM.pdf]

nature portfolio

Corresponding author(s): Baljit S. Khakh

Last updated by author(s): Jan 24, 2023

Reporting Summary

Nature Portfolio wishes to improve the reproducibility of the work that we publish. This form provides structure for consistency and transparency in reporting. For further information on Nature Portfolio policies, see our Editorial Policies and the Editorial Policy Checklist.

Statistics

For all statistical analyses, confirm that the following items are present in the figure legend, table legend, main text, or Methods section.

n/a	Confirmed	
☐	☒	The exact sample size (*n*) for each experimental group/condition, given as a discrete number and unit of measurement
☐	☒	A statement on whether measurements were taken from distinct samples or whether the same sample was measured repeatedly
☐	☒	The statistical test(s) used AND whether they are one- or two-sided<br>*Only common tests should be described solely by name; describe more complex techniques in the Methods section.*
☐	☒	A description of all covariates tested
☐	☒	A description of any assumptions or corrections, such as tests of normality and adjustment for multiple comparisons
☐	☒	A full description of the statistical parameters including central tendency (e.g. means) or other basic estimates (e.g. regression coefficient) AND variation (e.g. standard deviation) or associated estimates of uncertainty (e.g. confidence intervals)
☐	☒	For null hypothesis testing, the test statistic (e.g. *F*, *t*, *r*) with confidence intervals, effect sizes, degrees of freedom and *P* value noted<br>*Give P values as exact values whenever suitable.*
☒	☐	For Bayesian analysis, information on the choice of priors and Markov chain Monte Carlo settings
☒	☐	For hierarchical and complex designs, identification of the appropriate level for tests and full reporting of outcomes
☐	☒	Estimates of effect sizes (e.g. Cohen's *d*, Pearson's *r*), indicating how they were calculated

Our web collection on statistics for biologists contains articles on many of the points above.

Software and code

Policy information about availability of computer code

Data collection

Proteomics: The spectra were collected using data dependent acquisition on Orbitrap Fusion Lumos Tribrid mass spectrometer (Thermo Fisher Scientific) with an MS1 resolution of 120,000 followed by sequential MS2 scans at a resolution of 15,000. Data generated by LC-MS/MS were searched using the Andromeda search engine integrated into the MaxQuant (Cox et al,. 2008) bioinformatic pipelines against the Uniprot Mus musculus reference proteome (UP000000589 9606) and then filtered using a "decoy" database-estimated false discovery rate (FDR) < 1%. Label-free quantification (LFQ) was carried out by integrating the total extracted ion chromatogram (XIC) of peptide precursor ions from the MS1 scan. These LFQ intensity values were used for protein quantification across samples. Label-free quantification was carried out by the MaxQuant software with integrated search engine, Andromeda (https://www.maxquant.org/).
RNA-seq: Sequencing was performed on Illumina NextSeq 4000 for 2 x 75 yielding at least 45 million reads per sample. Demultiplexing was performed with Illumina Bcl2fastq2 v 2.17 program. Reads were aligned to the mouse mm10 reference genome using the STAR spliced read aligner (Dobin et al 2013)
Behavior: Open field and elevated plus maze behavior data including locomotor and anxiety behavior was collected and analyzed simultanously by Anymaze (Stoelting Co. Wooddale, IL, USA).
Imaging for IHC, ICC, RNA-scope, and proximity ligation assay was conducted on an Olympus FV3000 confocal microscope using Fluoview software.
Western blot data was collected on a GE Amersham 680 imager and on a Licor odyssey infrared imager.

Data analysis

Proteomics: Label-free quantification was carried out by the MaxQuant software with integrated search engine, Andromeda (https://www.maxquant.org/). Principal component data visualization was conducted with R package Factoextra fviz 1.0.6 (https://rpkgs.datanovia.com/factoextra/reference/fviz_pca.html). Differential protein expression and enrichment analysis was conducted with Bioconductor R package, limma v 3.54 (https://bioconductor.org/packages/release/bioc/html/limma.html). Protein network visualization, including STRING analysis was conducted with Cytoscape v 3.8 (https://apps.cytoscape.org/apps/stringapp). The artMS package v 1.16 (https://bioconductor.riken.jp/packages/3.8/bioc/html/artMS.html) was used to re-format the maxquant results (evidence.txt file), to make them compatible with SAINTexpress program. SAINT protein interaction probability scoring was done through (http://saint-apms.sourceforge.net/Main.html).

RNA-seq: Differential gene expression and enrichment analysis used R package limmaVoom v 3.36 to process RNA counts (https://rdrr.io/bioc/limma/man/voom.html) and batch correction was done with R package ComBat v 3.46 (https://rdrr.io/bioc/sva/man/ComBat.html).
IHC, ICC, RNA-scope, proximity ligation assay, western blots: Microscopy data and western blot data was imported and analyzed on FIJI (ImageJ v 2.1) using the BioFormats importer for Olympus FV3000 acquired images.
Behavior, IHC, ICC, RNA-scope, proximity ligase assay and Western data were plotted with OriginPro 2018 (v 9.6.5) and statistical analysis was conducted with GraphPad Instat 3.

For manuscripts utilizing custom algorithms or software that are central to the research but not yet described in published literature, software must be made available to editors and reviewers. We strongly encourage code deposition in a community repository (e.g. GitHub). See the Nature Portfolio guidelines for submitting code & software for further information.

Data

Policy information about availability of data

All manuscripts must include a data availability statement. This statement should provide the following information, where applicable:
- Accession codes, unique identifiers, or web links for publicly available datasets
- A description of any restrictions on data availability
- For clinical datasets or third party data, please ensure that the statement adheres to our policy

All the proteomic data are available at PRIDE with accession IDs PXD029257. The UniProt reference proteome used was UniProt UP000000589 9606 for Mus musculus. The RNA-seq data are available at GEO with accession ID GSE184773. All proteomic data are provided as supplemental information data tables 1-5. The analyzed RNA-seq data is provided as supplemental information table 6. All raw replicate data values used to generate the figures and the associated statistical tests are provided in supplemental information tables 7 and 8.

Field-specific reporting

Please select the one below that is the best fit for your research. If you are not sure, read the appropriate sections before making your selection.

☒ Life sciences ☐ Behavioural & social sciences ☐ Ecological, evolutionary & environmental sciences

For a reference copy of the document with all sections, see nature.com/documents/nr-reporting-summary-flat.pdf

Life sciences study design

All studies must disclose on these points even when the disclosure is negative.

Sample size	Power analysis was conducted using values for power of 0.8 or higher and alpha of 0.1 or lower and an estimated effect size based on pilot data. Furthermore, group sizes were selected based on data from the use of similar models by our laboratory.
Data exclusions	No data was excluded from this manuscript
Replication	To verify the reproducibility of the experimental findings, all data collection was done in multiple batches comprising at least four replicates. The proteomic data analyses was conducted from 4 independently processed batches that each contained 8 mice for each experimental group (in all cases). Behavioral data was conducted as the mice became available from the breeding colony and each experiment/recording was done in 2-3 batches containing between 3-6 mice per group. All experiments were successfully replicated.
Randomization	For proteomic experiments, mice were purchased from the supplier and each cage was randomly allocated to a group ad priori to AAV injections. For behavioral experiments, the mice were randomly allocated to a group as they became available and of age from the breeding colony in alternation.
Blinding	For the behavioral analyses, the investigators were blinded to group allocation during data collection, as numerical mouse IDs were the only identifier used. For analyses, the investigators were blinded to groups and mouse IDs by using the ImageJ/FIJI FIle Name Encrypter / Blind Analysis Tools plugin. Furthermore, behavioral experiments were run or analyzed by different people when possible.<br>For IHC, ICC, Proximity ligase assay analyses: all file names were randomized/encripted using the ImageJ/FIJI FIle Name Encrypter / Blind Analysis Tools plugin so investigators were blinded to all groups and identifiers. Because some of these experiments required use of mice used for behavior experiments, investigators were also blinded during data collection by sole use of mouse ID as identifier.

Reporting for specific materials, systems and methods

We require information from authors about some types of materials, experimental systems and methods used in many studies. Here, indicate whether each material, system or method listed is relevant to your study. If you are not sure if a list item applies to your research, read the appropriate section before selecting a response.

March 2021

Materials & experimental systems

n/a	Involved in the study
☐	☒ Antibodies
☐	☒ Eukaryotic cell lines
☒	☐ Palaeontology and archaeology
☐	☒ Animals and other organisms
☒	☐ Human research participants
☒	☐ Clinical data
☒	☐ Dual use research of concern

Methods

n/a	Involved in the study
☒	☐ ChIP-seq
☒	☐ Flow cytometry
☒	☐ MRI-based neuroimaging

Antibodies

Antibodies used

Primaries:
mouse anti-HA (Biolegend, 901514)
rabbit anti-HA (abcam ab9110)
rabbit anti-β-actin (Abcam, ab8227)
rabbit anti-S100β (Abcam, ab13970)
rabbit anti-NeuN (Cell Signaling, 12943S)
guinea pig anti-neuN (Synaptic Systems, 266004)
rabbit anti-DARPP32 (Abcam, ab40801)
guinea pig anti-DARPP32 (Frontier Institute, DARPP-Gp-A250)
chicken anti-GFP (Abcam, ab13970)
mouse anti-RFP (Rockland, 600906379)
rabbit anti-RFP (Rockland, 600401379)
guinea pig anti- RFP (Synaptic Systems, 390004)
rabbit anti-PAICS (Invitrogen, 92985)
mouse anti-Nebl (Santa Cruz Biotechnology, 393784)
rabbit anti-Slc4a4/NBC1 (Novus, NBP32020)
rabbit anti-Arpc1a (Invitrogen 102339)
rabbit anti-Faim2 (Origene, TP300196)
rabbit anti-Hepacam (Novus Biologicals, 04983)
mouse anti- APC (abcam, ab16794)
rabbit anti- Olig2 (Millipore, AB9610)
rabbit anti- ΔFosB (Cell Signaling Technology, 14695S)
mouse anti- Glt1 (Santa Cruz Biotechnology, sc-365634)
mouse anti- Ezrin (BioLegend, 866401)

Secondaries:
Streptavidin-HRP (Sigma, RABHRP3)
IR-dye 800CW anti-rabbit (Li-Cor, 929-08972)
Alexa Fluor 488 goat anti-chicken (Molecular probes, A11039)
Alexa Fluor 647 goat anti-rabbit (Molecular probes, A21244)
Alexa Fluor 546 goat anti-mouse (Molecular probes, A11003)
Alexa Fluor 488 goat anti-rabbit (Molecular probes, A11008)
Alexa Fluor 546 goat anti-mouse (Molecular probes, A11003)
Streptavidin, Alexa Fluor 488 conjugate (Molecular probes, S11223)
Donkey anti-guinea pig Cy3 (Jackson ImmunoResearch, 706-165-148)
Goat anti-rabbit plus 647 (Invitrogen, A32733)
Goat anti-chicken plus 555 (Invitrogen, A32932)

Validation

Most of the antibodies used in this manuscript have been validated and reproduced by our lab across at least 7 manuscripts by checking cell specificity, background signal, and noting antigen specificity using western blot techniques (Srinivasan et al., 2016; Chai et al., 2017, Nagai et al., 2019; Yu et al., 2020; Diaz-Castro et al., 2019; Endo et al., 2022, Gangwani et al., 2023). All Khakh lab manuscripts. The SAPAP3 antibody was validated extensively by Dr. Guoping Feng's group by IHC in mouse brain and co-corresponding in situ hybridization (Welch et al., 2005).

Anti-Nebl, anti-NBC1/Slc4a4, anti-Faim2, anti-Arpc1a, anti-Hepacam, and anti-PAICS were stated to be validated by the supplier.

Anti-Nebl was validated by Santa Cruz biotechnologies by western blot and immunofluorescence. Furthermore this antibody has been used in mouse tissue IHC as referenced by Rudolph et al., 2020.
Anti NBC/Slc4a4 was validated by Novus Biologicals by "orthogonal" strategies in which the antibody was tested by IHC in different types of tissue including a negative control tissue where there is no positivity as expected (lymph node) in correlation with RNA-seq to predict where positivity can occur.
Anti-Faim2 was validated by Origen in mouse tissue by both western blot and IHC techniques in mouse brain.
Anti-Arpc1a was validated by Invitrogen with western blot and immunofluorescence techniques in tissue.
Anti-HEPCAM was validated by Novus biologicals with western blots across different types of lysates including mouse brain.
Anti-PAICS was validated by Invitrogen with IHC, ICC, and western blots in rats, which have the same PAICS sequence as mouse.

Eukaryotic cell lines

Policy information about cell lines

Cell line source(s)	HEK 293T cells from ATCC (sex: female, RRID: CVCL_0045)
Authentication	The cells were authenticated by the supplier (ATCC) and by cell morphology.
Mycoplasma contamination	Cell line was not tested for mycoplasma
Commonly misidentified lines (See ICLAC register)	No misidentified cell lines were used in this study

Animals and other organisms

Policy information about studies involving animals; ARRIVE guidelines recommended for reporting animal research

Laboratory animals	Proteomics experiments for Figs 1-3 were conducted with 7 week old wild-type C57BL/6NTac mice from Taconic Biosciences. Both male and female mice were used in alternating batches. Behavioral experiments were conducted with 5-6 month old targeted knockout mice for Dlgap3 from Jackson Laboratories (B6.129-Dlgap3tm1Gfng/J). Both males and females were used in alternating batches.
Wild animals	The study did not involve wild animals
Field-collected samples	This study did not use field-collected samples
Ethics oversight	All experiments were conducted in accordance with the National Institutes of Health (NIH) Guide for the Care and Use of Laboratory Animals and were approved and overseen by the Chancellor's Animal Research Committee (ARC) at the University of California, Los Angeles (UCLA)

Note that full information on the approval of the study protocol must also be provided in the manuscript.
