# Supplementary material for: Astrocyte–neuron subproteomes and obsessive–compulsive disorder mechanisms
Source: Nature. 2023 Apr 12;616(7958):764–73. doi: 10.1038/s41586-023-05927-7 (PMC10132990; doi:10.1038/s41586-023-05927-7)
Supplement: Supplementary file 12 — New AAV constructs and Addgene identifiers. [file 41586_2023_5927_MOESM12_ESM.pdf]

| AV construct | Addgene ID | Titer |
|---|---|---|
| *AAV2/5 *GfaABC₁D* BioID2-Linker-BioID2-HA | 176740 | 3.7 x $10^{13}$ gc/ml |
| *AAV2/5 *GfaABC₁D* Lck-BioID2-Linker-BioID2-HA | 176741 | 3.1 x $10^{13}$ gc/ml |
| *AAV2/5 *GfaABC₁D* Aqp4-BioID2-Linker-BioID2-HA | 176742 | 1.1 x $10^{13}$ gc/ml |
| *AAV2/5 *GfaABC₁D* Cx43-BioID2-Linker-BioID2-HA | 176743 | 2.5 x $10^{13}$ gc/ml |
| *AAV2/5 *GfaABC₁D* Ezr-BioID2-Linker-BioID2-HA | 176744 | 1.0 x $10^{13}$ gc/ml |
| *AAV2/5 *GfaABC₁D* Glt1-BioID2-Linker-BioID2-HA | 176745 | 2.0 x $10^{13}$ gc/ml |
| *AAV2/5 *GfaABC₁D* HA-BioID2-Linker-BioID2-Kir4.1 | 176746 | 6.3 x $10^{13}$ gc/ml |
| AAV2/5 *GfaABC₁D* tdTomato | 44332 | 1.4 x $10^{13}$ gc/ml |
| AAV2/5 *GfaABC₁D* Lck-eGFP | 52925 | 1.5 x $10^{13}$ gc/ml |
| *AAV2/5 *GfaABC₁D* eGFP | 176861 | 1.1 x $10^{13}$ gc/ml |
| *AAV2/5 *GfaABC₁D* Aqp4-eGFP | 176747 | 2.4 x $10^{13}$ gc/ml |
| *AAV2/5 *GfaABC₁D* Cx43-eGFP | 176860 | 1.4 x $10^{13}$ gc/ml |
| *AAV2/5 *GfaABC₁D* Ezr-eGFP | 176856 | 1.6 x $10^{13}$ gc/ml |
| *AAV2/5 *GfaABC₁D* Glt1-eGFP | 176862 | 5.8 x $10^{13}$ gc/ml |
| AAV2/5 *GfaABC₁D* eGFP-Kir4.1 | 52874 | 2.7 x $10^{12}$ gc/ml |
| *AAV5 *GfaABC₁D* eGFP-SAPAP3 | 176859 | 1.0 x $10^{13}$ gc/ml |
| *AAV5 *GfaABC₁D* HA-BioID2-SAPAP3 | 176858 | 2.4 x $10^{13}$ gc/ml |
| AAV2/5 *GfaABC₁D* Rpl22-HA | 111811 | 2.1 x $10^{13}$ gc/ml |
| *AAV5 *GfaABC₁D* HA-SAPAP3 | 190200 | 1.0 x $10^{13}$ gc/ml |
| *AAV5 *GfaABC₁D* LifeAct-eGFP | 190199 | 1.0 x $10^{13}$ gc/ml |
| *AAV1 *hSyn1* HA-BioID2-SAPAP3 | 176857 | 2.0 x $10^{13}$ gc/ml |
| *AAV1 *hSyn1* eGFP-SAPAP3 | 176853 | 2.0 x $10^{13}$ gc/ml |
| *AAV5 *hSyn1* Rpl22-HA | 177685 | 1.0 x $10^{13}$ gc/ml |
| *AAV1 *hSyn1* eGFP | 50465 | 1.3 x $10^{13}$ gc/ml |

**Supplementary Table 9.** Plasmids used in this study (* new AAV plasmids generated in this study).
